# Microglial TIR-domain-containing adapter-inducing interferon-β (TRIF) deficiency promotes retinal ganglion cell survival and axon regeneration via nuclear factor-κB

**DOI:** 10.1186/1742-2094-9-39

**Published:** 2012-02-24

**Authors:** Sen Lin, Yajie Liang, Jiqiang Zhang, Chen Bian, Hongli Zhou, Qiang Guo, Ying Xiong, Shurong Li, Bingyin Su

**Affiliations:** 1 Department of Neurobiology, Chongqing Key Laboratory of Neurobiology, Third Military Medical University, Chongqing 400038, PR China; 2 Department of Histology and Embryology and Neurobiology, Development and Regeneration Key Laboratory of Sichuan Province, Chengdu Medical College, Chengdu 610083, PR China; 3 Department of Pathology, Chengdu Medical College, Chengdu 610083, PR China

**Keywords:** TRIF, Optic nerve, Retinal ganglion cell, Microglial cell, Inflammation

## Abstract

**Background:**

TIR-domain-containing adapter-inducing interferon-β (TRIF) is the sole downstream adaptor of Toll-like receptor (TLR)3, which is one of the major signaling pathways in immune cells leading to neuroinflammation in the central nervous system. Overexpression of TRIF may lead to activation of inflammatory responses, and contribute to pathophysiological progression in both acute and chronic neurodegenerative retinal diseases. In the present study, was aimed to elucidate the contributions of TRIF to optic nerve (ON) regeneration and retinal ganglion cell (RGC) survival following injury to the ON, a widely studied model of central nervous system injury and of degenerative diseases such as glaucoma.

**Methods:**

We used retrograde labeling with a fluorochrome, hydroxystilbamidine (Fluorogold) to evaluate RGC survival, and immunostaining with growth-associated protein-43 to evaluate axon regeneration in an ON crush model. Changes in microglial cytokines following RGC injury was examined with ELISA and real-time PCR. *In vivo *studies were carried out in wild-type and *trif^-/- ^*mice. A Transwell co-culture system and migration test were used to mimic the crosstalk between microglia and RGCs. TRIF-associated downstream adaptors were determined by western blotting.

**Results:**

Compared with wild-type (WT) mice, TRIF knockout (KO) mice displayed a robust ability to regenerate axons 3 or 7 days after nerve injury. In addition, RGC survival was considerably higher in *trif^-/- ^*than in WT mice. ON lesion induced less microglial activation in *trif^-/- ^*than in WT mice. and more WT microglia distorted and migrated toward the foramen opticum. In the transwell system, few *trif^-/- ^*microglia migrated through the membrane when stimulated by the performed lesion on RGC axons in a transwell system. Inactivation of microglial cells in *trif^-/- ^*mice was associated with reduced production of inflammatory cytokines, as detected with real-time RT-PCR and ELISA. Furthermore western blot analysis showed that activation of known downstream effectors of TRIF, including TBK1, IKKε and NF-κB, were significantly inhibited by TRIF deficiency.

**Conclusion:**

Our results indicate that TRIF deficiency promotes ON axon regeneration by attenuating microglial activation and consequently reducing the release of harmful cytokines via NF-κB inactivation.

## Background

Axon regeneration in the central nervous system (CNS) is limited by both cell-intrinsic and environmental inhibitory molecules [1-4]. The optic nerve (ON) crush model is considered to be a classic model for studying CNS regeneration [4-9]. Microglia act as tissue macrophages in the CNS, thus they play a role in tissue maintenance and immune surveillance [10], and become activated under pathological conditions, including neurodegenerative diseases and neural injury [11,12]. There is increasing evidence that inflammatory factors, such as interleukins (ILs), tumor necrosis factor (TNF)-α, and nitric oxide (NO), released by activated or over-activated microglial cells [13-15], affect neural cell survival [1,16]. Pro-inflammatory cytokines are produced largely in response to Toll-like receptor (TLR) activation in microglial cells.

In the CNS, TLRs are mainly found on immune cells, such as microglia and macrophages [17-20]. An alternative downstream adaptor, TRIF, is recognized as the sole transducing signal in the TLR3 signaling pathway in response to double-stranded RNA. The TLR4 signaling pathway acts via a myeloid differentiation factor (MyD)88 -independent pathway, leading to the subsequent activation of nuclear factor (NF)-κB and interferon regulatory factor (IRF)3, which induces interferon (IFN)-β release [21]. In an ischemia-reperfusion model, we previously found that high mobility group protein (HMG)B1 mediates injury via TRIF-independent TLR4 signaling [22]. However, the involvement of MyD88 or TRIF may differ in different tissues and cells. Dual signaling of MyD88 and TRIF is crucial for dendritic cell maturation [23]. The TLR3/TRIF signaling pathway is required for apoptosis of melanoma cells by polyinosinic-polycytidylic acid (poly I:C)-induced activation of caspase-8 [24]. TLR3/TRIF/receptor-interacting protein (RIP)1 signaling is also essential for human airway epithelial cells apoptosis via caspase-mediated activation [25]. However, the role of TRIF in neural apoptosis and axonal degeneration/regeneration remains unclear. The current study was designed to determine the potential role of TRIF in ON injury and retinal ganglion cell (RGC) survival, and the downstream mechanisms involved.

We found that *trif^-/- ^*mice exhibit increased retinal axon regeneration and less RGC loss compared with wild-type (WT) mice. Our results indicate that TRIF deficiency attenuates microglial activation and downstream signaling, and limits the release of inflammatory cytokines following ON injury.

## Methods

### Animals

All animal-related procedures in this study were in strict accordance with the Third Military Medical University (TMMU) guidelines for the use of experimental animals. The Animal Ethics Committee of TMMU approved all experimental procedures used in the present study.

SPF grade adult male C57BL/6 male mice (20-24 g) (Animal Center, Third Military Medical University, Chongqing, China), and male *trif^-/- ^*mice (C57BL/6 J-*AW046014^Lps2^*/J; (Jackson Laboratory, Bar Harbor, ME, USA), aged 8-10 weeks (20-24 g) of age, were used. All mice were housed on a 12 hour light/dark schedule with water and food available *ad libitum*. Neonatal C57BL/6 mice were used to make primary microglial cultures.

### Optic nerve crush model

The ON is considered a classic model of CNS regeneration to investigate injury [4,6-9]. ON crush was carried out as described previously [7]. In brief, adult WT C57BL/6 mice and *trif^-/- ^*mice were anesthetized with intraperitoneal (IP) injection of chloral hydrate in PBS (400 mg/kg), and the ON was crushed as described [4,6,7]. Animals with permanent ischemia were excluded. All procedures were performed aseptically and on the left eye, with the right eye serving as a sham-operated control.

### Fixation and sectioning

Animals were killed at the end of the treatment period with intraperitoneal injection of chloral hydrate in PBS, perfused through the heart with 0.9% saline, followed by 4% paraformaldehyde (PFA). The eyes were removed and post-fixed in 4% PFA for 4 hours at 4°C, and then incubated in 30% sucrose overnight at 4°C. The eye cups and ONs were cryosectioned into slices 15 μm thick on a rapid sectioning cryostat (CM 1900 Leica and thaw-mounted onto coated glass glides (Superfrost Plus, Fisher, Pittsburgh, PA, USA).

### Retinal ganglion cell and microglial cell culture

RGCs were purified from the retinas of *trif^-/- ^*and WT mice on post-natal day 1 by immunopanning, as previously described [26]. Axon outgrowth and cell survival in serum-free DMEM (Sigma, St. Louis, MO, USA) were assessed after maintaining the plate at 37°C for 3 days. As previously described [6], axon growth was defined as the percentage of RGCs that extended axons of no less than two cell diameters in length.

For microglia culture, the cortex of the cerebral hemispheres of 1-day-old post-natal mice were dissected, and digested with 0.125% trypsin. After centrifugation for 5 minutes at 300 × g, the lower precipitation products were seeded onto a six-well plate pre-coated with poly-L-lysine, and incubated with DMEM and 10% FBS (Hyclone, Logan, UT, USA). Culture medium was refreshed twice a week for 2 weeks, and the microglia were detached by mild shaking, then filtered through a nylon mesh to remove astrocytes. After centrifugation at 300 × g for 5 minutes, the cells were resuspended in fresh DMEM supplemented with 10% FBS and plated at a final density of 5 × 10^5^/ml cells on a poly-L-lysine pre-coated six-well culture plate. Cell purity was determined by immunohistochemical staining with microglia-specific antibodies for CD11b and F4/80, and purity was determined to be > 90%.

### Antibodies and immunofluorescence staining

Tissue sections were rinsed in 0.01 mol/l PBS, and then incubated in 5% normal donkey serum diluted in PBS for 1 hour at 25°C. Following removal of serum, tissue sections were incubated overnight with primary antibodies. An antibody to growth-associated protein (GAP)43 was used to label regenerated axons within the ON (1:1000, sheep anti-mouse; supplied by Prof. Y. Yin). Rabbit polyclonal antibody to TRIF (1:200 dilution; catalogue number ab13810; Abcam, Cambridge, Cambridgeshire, UK) was used to visualize TRIF. CD11b (1:200; 14-0112-81; eBioscience, San Diego, CA, USA) and Iba-1 (1:50; 15690; Abcam) were used as a marker for microglia. On the second day, the sections were washed in PBS and then incubated with secondary antibody for 1 hour at 25°C. Fluorescent secondary antibodies were used to visualize the primary antibody staining: goat anti-rat Alexa Fluor^® ^488 (A11006, 1:300), goat anti-rabbit Alexa Fluor^® ^568 (A11011, 1:300), and donkey anti-sheep Alexa Fluor^® ^568 (A21099, 1:300) all Invitrogen Corp., Carlsbad, CA, USA). Sections incubated with preimmune rabbit IgG (17-615, Millipore, USA) served as a negative control. After washing with PBS, sections were stained with 4', 6-diamidino-2-phenylindole (DAPI, 0.5 μg/ml) for 10 minutes at 25°C and then rinsed with PBS, and mounted with a fluorescent mounting medium (DAKO Cytomation, Glostrup, Denmark). Co-localization of TRIF and IBA1 were examined under a confocal microscope (LSM FV1000; Olympus Company Pte Ltd, Tokyo, Japan).

For retinal flat-mounts, eyes were removed and post-fixed in 4% PFA for 30 minutes. Retinas from the intact right eyes of the same animals were used as normal controls. After three washes in PBS, retinas were blocked and permeabilized using 5% goat serum (Hunter Antisera, New South Wales, Australia) and 0.2% Triton X-100 for 1 hour at 25°C, and then incubated with CD11b (1:200; 14-0112-81; eBioscience) and a βIII-tubulin antibody (1:500; Babco, Richmond, CA, USA) for 2 days at 4°C. The next day, retinas were rinsed with PBS, then incubated with a goat anti-rabbit Alexa Fluor^® ^568 secondary antibody (1:300, Invitrogen) at 4°C overnight, rinsed again, and overlaid with a coverslip in mounting medium.

Cells were fixed with 4% PFA at 25°C for 30 minutes, then blocked with 5% bovine serum albumin (BSA, Sigma-Aldrich, St Louis, MO, USA) for 30 minutes at 25°C. The cells were incubated with primary antibody (GAP43, 1:1000) for 1 hour at 25°C, followed by overnight incubation at 4°C. The next day, cells were exposed to secondary antibody (Alexa Fluor^® ^568-conjugated donkey anti-sheep IgG (H + L), 2 mg⁄mL, Invitrogen) for 1 hour at 25°C. Axon outgrowth was evaluated in quadruplicate samples (~20 RGCs per well) in a blinded fashion, and all experiments were repeated at least three times, independently.

### Retinal ganglion cell axon retrograde labeling

WT and *trif^-/- ^*male mice were anesthetized and placed in a stereotactic apparatus (Stoelting, Kiel, WI, USA). The skull was exposed and cleaned with 3% hydrogen peroxide. A hole 1 mm in diameter was drilled in the skull (4.0 mm posterior and 0.06 mm lateral to the bregma), and a 26-gauge stainless steel cannula was inserted for infusion of a fluorochrome, hydroxystilbamidine (Fluorogold; Biotium, Inc., Hayward, CA, USA) infusion (1 μl/10 min). One week before ON lesion. 1 μl of 4% Fluorogold (FG) was injected into the bilateral superior colliculus (1.2 mm deep from the skull)

### Analysis of axon regeneration and Fluorogold-labeled retinal ganglion cells

Analysis of axon regeneration and RGC survival were conducted in accordance with a previous report [6]. Briefly, regenerating axons were examined using a calibrated ocular to measure distance in five longitudinal sections of the ON by GAP43 immunostaining; 8 to 10 sections per animal were used in the quantification. A researcher blinded to the sample identity quantified axon growth by counting the total number of GAP43-positive axons arising from RGCs at various distances past the lesion site (100, 200, 300, 400, and 500 μm from the end of the crush site). The calculation of axon quantification was conducted in accordance with the method of Yin [4]. Axon counts were converted into axon crossings (axons/mm), and the mean over the five sections was calculated. Σa_*d*_, defined as the total number of axons extending distance *d *in a optic nerve with a radius of *r*, was estimated by summing over all sections of thickness *t *as follows:

$$\sum a_{d}=\pi {\text{r}}^{2}\times \left(average\;axons/mm\;width\right)/t$$

Total axon number was calculated in each case. Analysis of variance (ANOVA) was used to test the significance of the differences between groups. To analyze the survival number of RGCs in whole retinas labeled with FG at 0, 1, 3, and 7 day post-crush (dPC), the gold dots (surviving RGCs) were counted using Image Pro Plus (version 6.0, Media Cybernetics, Inc., Bethesda, MD, USA).

### Western blotting

For cultured microglia or neurons, cells were washed in sterile PBS, then lysed in 2% SDS (in deionized water) with a protease inhibitor cocktail (118836153001; Roche Diagnostics, Indianapolis, IA, USA) at a concentration of 1 × 10^6 ^cells/mL. The lysate was then separated by centrifugation at 12000 *g *at 4°C for 15 minutes. The supernatant was collected and the protein concentration was measured using a bicinchoninic acid protein assay (Pierce, Rockford, IL, USA); 35 μg samples were loaded into 8% SDS-polyacrylamide gels. Proteins were then transferred to polyvinylidene difluoride membranes (Millipore Corporation, Bedford, MA, USA) using a 100 V current for 1.5 hours. The blots were then first washed with Tris-buffered saline and Tween (TBS-T; 50 mmol/L Tris pH 7.4, 150 mmol/L NaCl, and 0.1%Tween), followed by blocking in 5% non-fat milk-TBS-T overnight at 4°C. Antibodies recognizing NF-κB (1:500, AN365, Beyotime, China), TANK-binding kinase (TBK)1 (1:500; ab40676; Abcam, Cambridge, UK), IκB kinase (IKK)ε (1:1000; LS-B63; Lifespan, Seattle, WA, USA), and GAP43 (a gift from Yin [4]) were made up in a solution of in 5% milk in TBS-T, and used overnight at 4°C, followed by three washes with TBS-T and incubation with horseradish peroxidase-conjugated anti-rabbit, anti-sheep or anti-rat IgG secondary antibodies (Santa Cruz Biotechnology Inc., Santa Cruz, CA, USA) in TBS-T for 1.5 hours at 25°C. The blot was developed with DAB and a commercial chemiluminescent detection system (SuperSignal^® ^West Pico Chemiluminescent Substrate Detection System; Thermo Fisher Scientific Inc., Rockford, IL, USA).

### Tissue collection and cytokine measurement

#### Real-time reverse transcriptase PCR analysis

To analyze the mRNA expression of cytokines, total RNA extraction and real-time PCR were performed as previously reported, with minor modifications [27]. Total RNA was extracted (RNA IsoPlus, TaKaRa Biotechnology Co. Ltd, Dalian, China) with 800 μl of the RNA lysis buffer supplied with the kit. RNA was reverse-transcribed (TaKaRa PrimeScript RT Reagent Kit; TaKaRa Biotechnology Co. Ltd) in accordance with the manufacturer's instructions. First-strand cDNAs were amplified using a real-time PCR thermal cycler (IQ5; Bio-Rad Laboratories, Inc., Hercules, CA, USA). Quantificative real-time PCR was performed with Taq polymerase (SYBR Premix Ex Taq II; TaKaRa Biotechnology Co. Ltd) in accordance with the manufacturer's instructions. Primers for IFN-β, β-actin, TNF-α, inducible NO synthase (iNOS), IL-1β, IL-6, and IL-17 (Sangon Biotech, Shanghai, China) are shown in Table 1. For relative comparison of each gene, we analyzed the cycle of threshold (Ct) value of real-time PCR data using the ^-ΔΔ^Ct method, in accordance with the company's instructions.

**Table 1:** Primers used in the experiments

Gene	Ref. Sequences Accession	Forward (5'-3')	Reverse (5'-3')
β-actin	NM_007393.3	AGATTACTGCTCTGGCTCCTAGC	ACTCATCGTACTCCTGCTTGCT

IL-1β	NM_008361.3	TCCAGGATGAGGACAT	GAACGTCACACACCAGCAGGTTA

IL-6	NM_031168.1	GAGGATACCACTCCCAACAGACC	AAGTGCATCATCGTTGTTCATACA

IL-17	NM_010552.3	ACGCGCAAACATGAGTCCAG	AGGCTCAGCAGCAGCAACAG

iNOS	NM_010927.3	AATTCGGCTGTGCTTTGATGG	GACTTGCGGGAGTCAGAATAGGAG

IFN-β	NM_010510.1	AAGCAGCTCCAGCTCCAAGAA	TTGAAGTCCGCCCTGTAGGTG

TNF-α	NM_013693.2	TCCAGGCGGTGCCTATGT	CGATCACCCCGAAGTTCAGTA

IFN, interferon; IL, interleukin; iNOS, inducible NO synthase; TNF, tumor necrosis factor.

#### ELISA analysis

Microglial cells were collected at 12, 24, and 36 hours after stimulation of injured RGCs. The cells were rinsed twice with PBS, and then lysed with a protease inhibitor cocktail (Roche Complete, Roche Diagnostics, Mannheim, Germany), and frozen at -80°C until analysis. For protein isolation, the samples were milled and separated by centrifugation at 10, 621 × g at 4°C for 10 minutes. The supernatant was carefully pipetted into a fresh 1.5 ml EP Eppendorf tube, and the protein concentration was evaluated by protein assay (BCA Protein Assay Kit; Beyotime, China). For TNF-α, IFN-β, IL-1β IL-6, and IL-17 detection, a mouse ELISA kit (R&D Systems, Minneapolis, MN, US) was used, in accordance with the manufacturer's instructions. Briefly, the plate was incubated with 100 μl of each sample or standard protein, in duplicate. After incubation and subsequent washing, horseradish peroxidase-conjugated streptavidin at 400 ng/ml detection antibody was added, followed by washing and incubation with the substrate solution provided with the kit to produce a color reaction, which was stopped by addition of stop solution. The absorbance was read at 450 nm in a microplate reader.

### Statistical analysis

**Figure 1:**
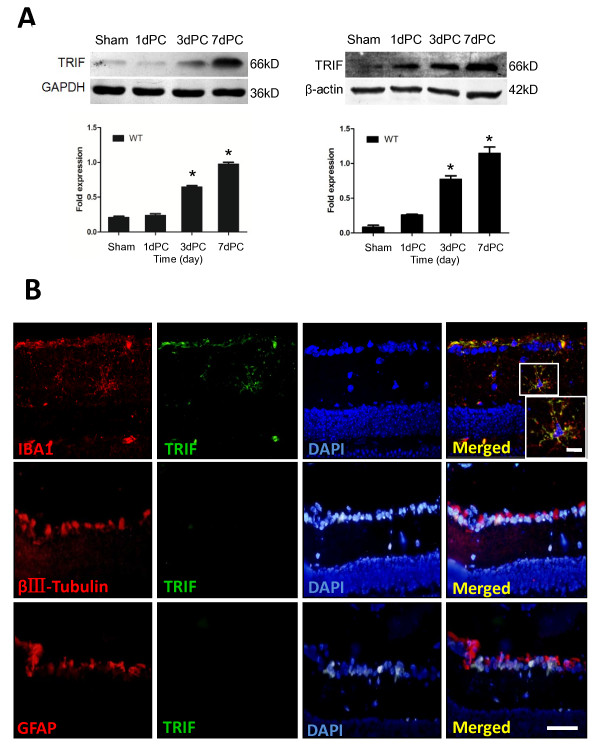
**TIR-domain-containing adapter-inducing interferon-β (TRIF) is expressed by microglia, but not by astrocytes or neurons, and is expressed in a time-dependent manner**. (**A**) Retinas were collected from sham-operated mice and from TRIF-deficient mice at 1, 3, and 7 days post-crush (1dPC, 3dPC, and 7dPC, respectively). TRIF expression was examined by western blotting, which showed that in the sham-operated and 1dPC groups, TRIF expression was limited. At 3dPC and 7dPC, TRIF expression was upregulated. GAPDH and β-actin were used as internal controls. *P < 0.05, increase relative to sham control. (**B**) Using dual-label immunofluorescence staining, expression of TRIF was detected in microglia but not neurons and astroglia. Iba-1, βIII-tubulin and glial fibrillary acidic protein (GFAP) were used to identify microglia, neurons, and astroglia in retinal sections. Scale bar = 20 μm. Scale bar (in box) = 10 μm.

**Figure 2:**
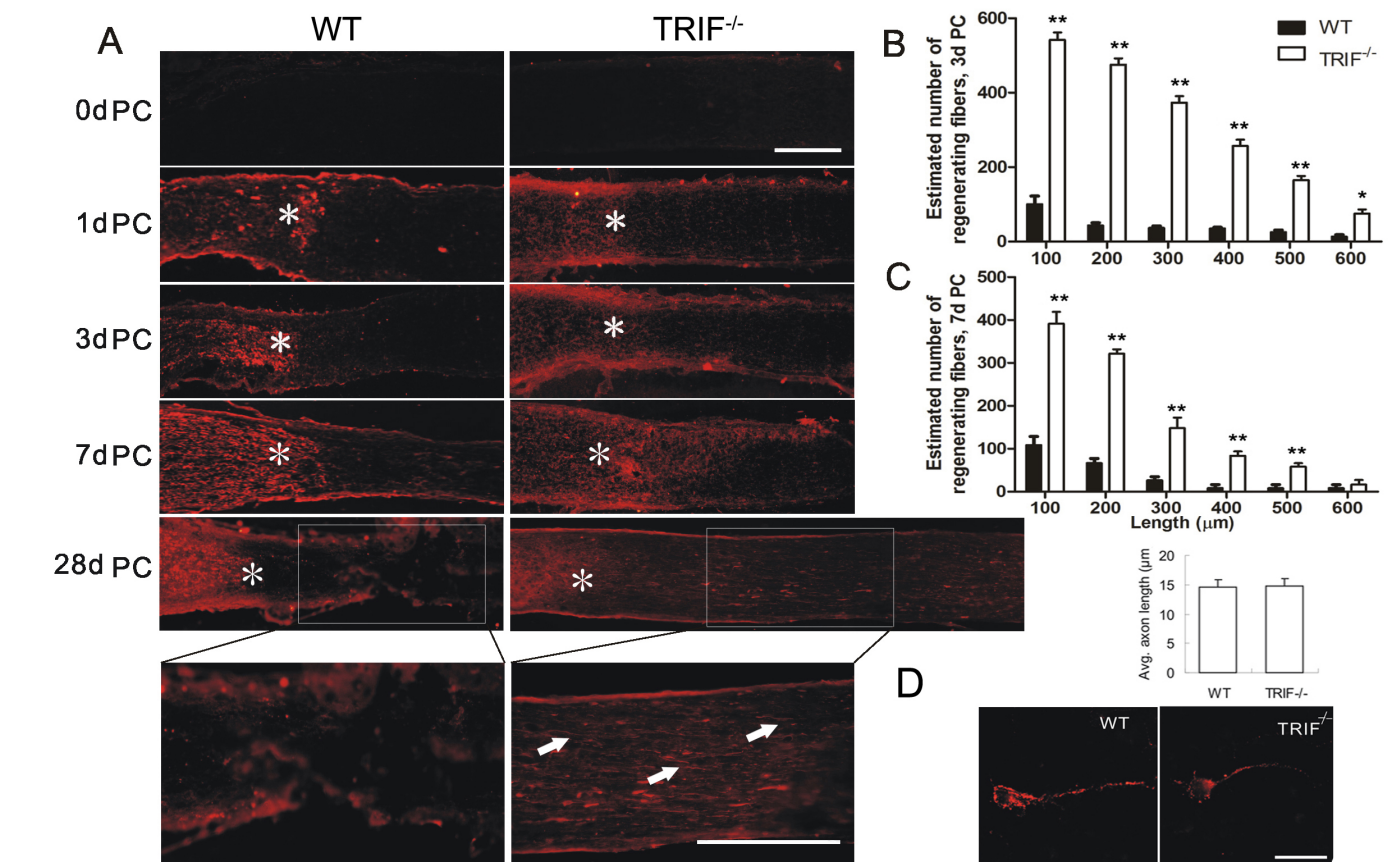
**TIR-domain-containing adapter-inducing interferon-β deletion promotes optic nerve regeneration**. (A) Longitudinal sections through an optic nerve immunostained to detect growth-associated protein43-positive axons distal to the injury site (*) at 0, 1, 3, 7 and 28 days after nerve crush. The *trif^-/- ^*mice exerted more robust regeneration ability than the wild-type (WT) mice. In WT mice, few axons were regenerated beyond the lesion site (*), in contrast to the *trif^-/- ^*group, in which regeneration was particularly marked at 28 days post-crush (28dPC). Upper scale bar = 100 μm, bottom scale bar = 50 μm. (**B**) Estimated number of regenerated fibers (> 100, 200, 300, 400, and 500 μm from the lesion site) at 3dPC and (C) 7dPC in the WT and *trif^-/- ^*groups. *P < 0.05, **P < 0.01, increase relative to WT. (D) Quantification of outgrown axons of *in vitro *retinal ganglion cells (RGCs) by GAP43 immunostaining. Axon length was calculated by two observers using a double-blinded method, under a microscope. Mean length of axons was measured using a micrometer.

**Figure 3:**
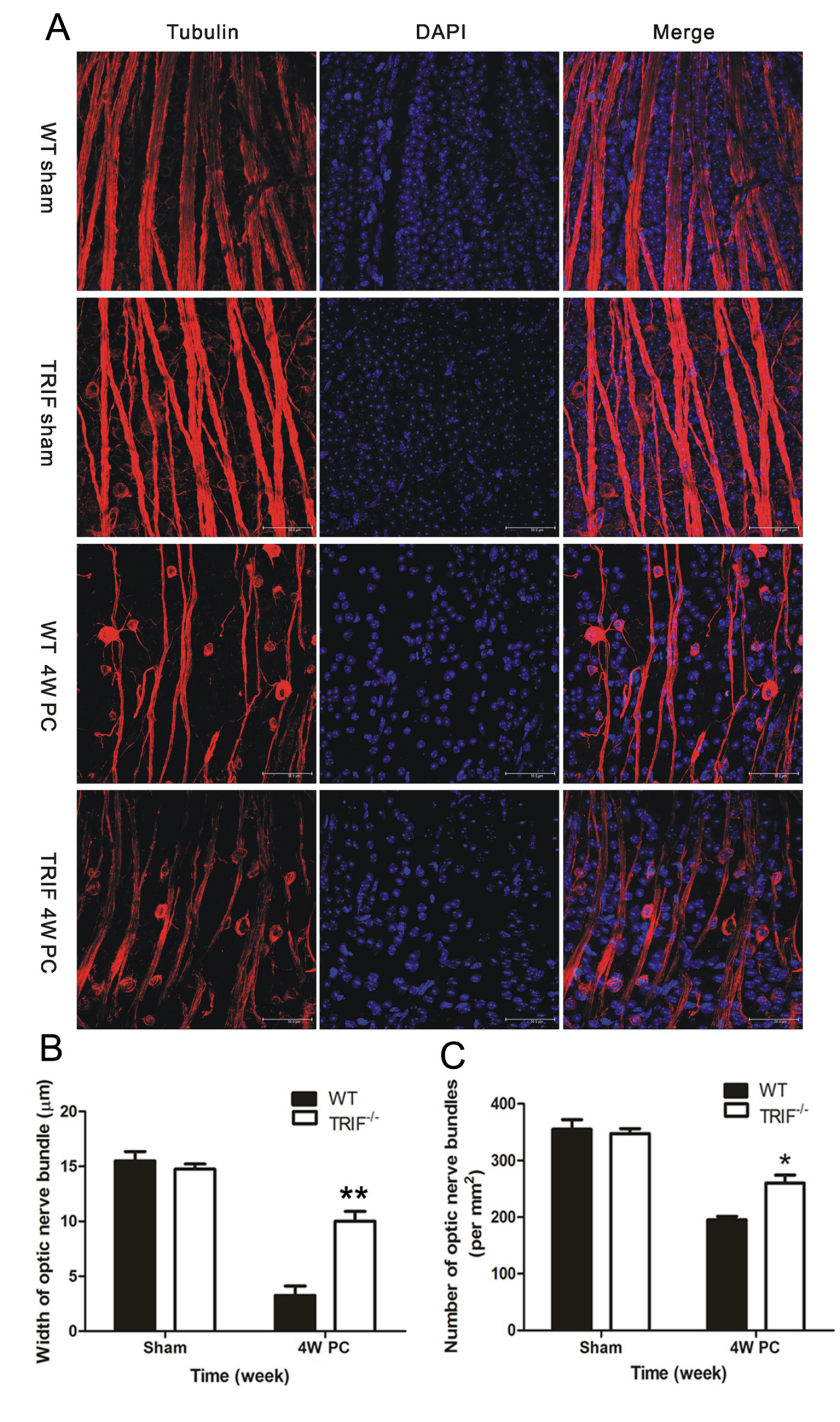
**Optic nerve bundles in whole-mount retina by βIII-tubulin immunostaining**. (**A**) Optic nerve bundles (ONBs) were immunostained with βIII-tubulin and DAPI. In both the WT and *trif^-/- ^*sham groups, ONBs displayed a regular and full shape. At 4 weeks post-crush (4wPC), both WT and *trif^-/- ^*ONB became attenuated; this was especially marked in the WT retina, which seems atrophic. Using double-blinded quantification, (**B**) WT retina were found to display narrower ONBs (B, n = 26) and (**C**) had a lower value (*ρ*_ONB_; n = 30) compared with those in the *trif^-/- ^*group.*P < 0.05, **P < 0.01, Scale bar = 50 μm.

**Figure 4:**
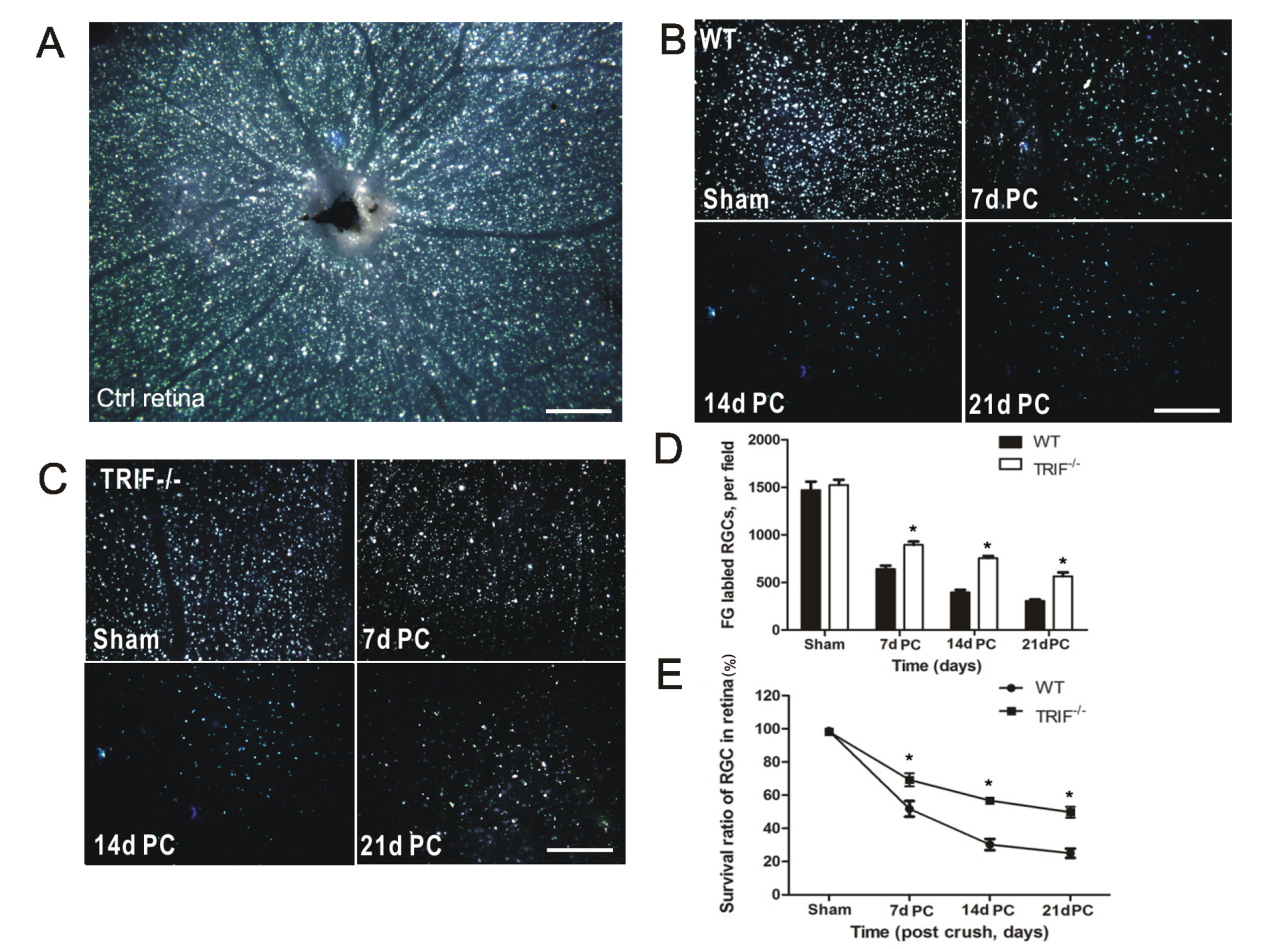
**Retinal retinal ganglion cell (RGC) survival detected by Fluorogold (FG) retrograde labeling**. (**A**) FG was transported retrograde to RGC soma in a whole-mount retina used as a control. From 7 to 21 days post-crush (7dPC to 21dPC, FG-labeled RGCs declined in (**B**) the WT and (**C**) *trif^-/- ^*groups. Scale bar, 100 μm. The number of labeled RGCs was analyzed to confirm that more RGCs survived in the *trif^-/- ^*group than in the WT group (**D**). *P < 0.05 vs. WT group. (**E**) Survival ratio of RGCs in *trif^-/- ^*group was higher than that in the WT group from 7dPC to 21dPC. *P < 0.05 vs. WT group.

**Figure 5:**
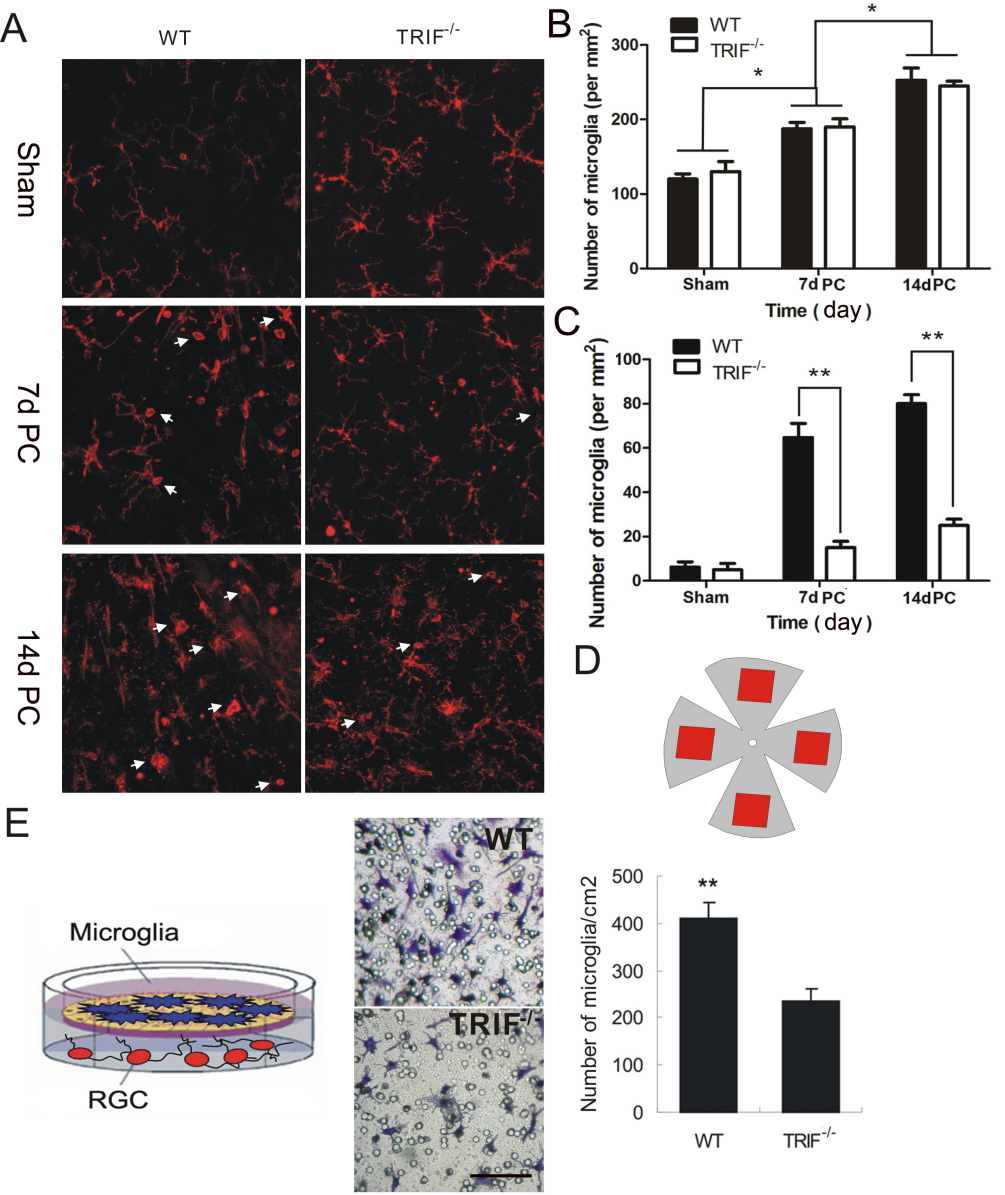
**Microglial activation in whole-mount retina and microglial migration in an *in vitro *model**. (**A**) Microglia in wild-type (WT) retina have a different shape from those in the *trif^-/- ^*group as shown by CD11b immunostaining at 7 and 14 days post-crush (7dPC and 14dPC). Scale bar = 20 μm. (**B**) Estimated numbers of microglia/mm^2 ^in whole-mount retinas were different between different time-course groups (*P < 0.05 increase relative to sham and 14dPC group). (**C**) Estimated number per mm^2 ^of microglia with an amoeboid shape in whole-mount retinas were different between wild-type (WT and *trif^-/- ^*groups at 7 and 14 days post-crush (7dPC and 14dPC). *P < 0.01 increase relative to WT group in 7dPC and 14dPC. (**D**) Outline of whole-mount retina. Square frames (red) were counted using double-blinded quantification. (**E**) In a transwell system, microglia and retinal ganglion cells (RGCs) were cultured together (as shown in drawing). Microglia became activated and migrated to the other side of the membrane when stimulated by injured RGCs in the lower well. Stained with cresyl violet acetate. Scale bar = 20 μm.

**Figure 6:**
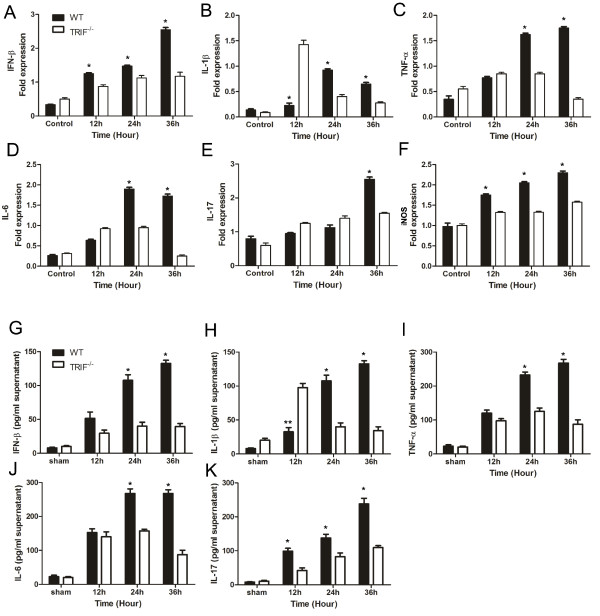
**TIR-domain-containing adapter-inducing interferon-&#946 (TRIF) deficiency attenuates inflammation via TANK-binding kinase (TBK)1/I&#954B kinase (IKK)&#949 and nuclear factor (NF)-&#954B signaling**. Western blot results for wild-type (WT) and trif-/- microglia pre-stimulated by injured retinal ganglion cells (RGCs) in a transwell system identifies the signaling changes downstream of TRIF. **(A)** Bar graph showing that TBK1 was upregulated gradually in the WT group; however, trif-/- effectively suppressed TBK1 from 24 to 36 hours. **(B)** Trif-/- effectively suppressed NF-&#954B from 12 to 36 hours. **(C)** Bar graph showing that trif-/- effectively suppressed IKK&#949 from 12 to 36 hours. *P <0.05, **P <0.01 vs. WT group at the same time point. &#946-Actin was used as an internal control.

**Figure 7:**
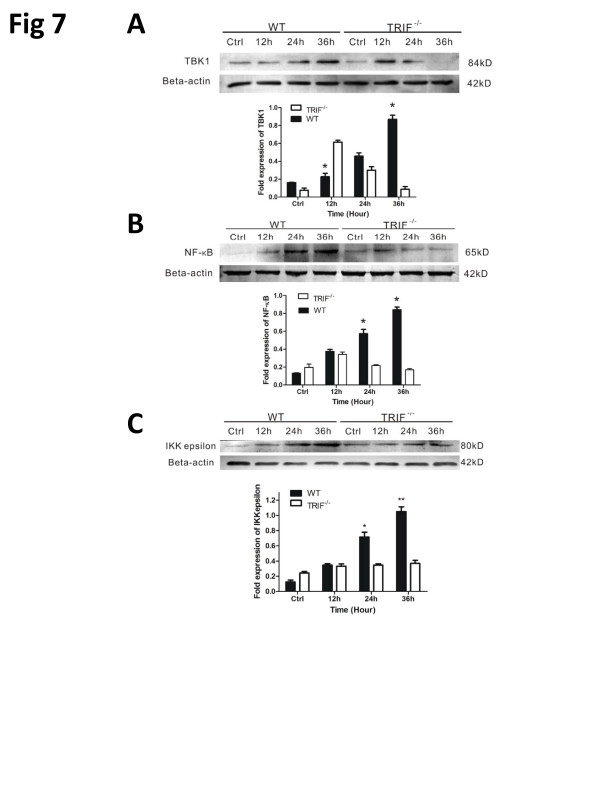
**TIR-domain-containing adapter-inducing interferon-β (TRIF) deficiency attenuates microglial inflammatory factor release**. Real-time reverse transcriptase (RT)-PCR and ELISA results of wild-type (WT) and *trif^-/- ^*microglia pre-stimulated by injured RGCs in a transwell system to identify changes of inflammatory factors. (**A-F**) Measured by real-time RT PCR; **(G-K) **measured by ELISA. (**A**) Bar graphs showing interferon (IFN)-β mRNA expressed in the control, and at 12, 24, and 36 hours in WT and *trif^-/- ^*microglia by real-time RT-PCR. Significant differences were seen at 36 hours in microglia pre-stimulated with injured RGCs. (**B**) Interleukin (IL)-1β mRNA expressed in the WT and *trif^-/- ^*groups. At 12 hours, IL-1β mRNA expression was higher in the *trif^-/- ^*than in the WT group; however, at 24 and 36 hours, it was higher in the WT than in the *trif^-/- ^*group. (**C**) At 24 hours and 36 hours, tumor necrosis factor (TNF)-α was upregulated to a greater extent in the WT group. (**D**) IL-6 was significantly higher in the WT than in the *trif^-/- ^*group at 24 and 36 hours. (**E**) IL-17 was upregulated at 36 hours in the WT. (**F**) Inducible nitric oxide synthase (iNOS) increased from 12 to 36 hours in the WT. Similar to the PCR results, (**G**) IFN-β release increased markedly at 24 and 36 hours. (H) IL-1β had a greater increase at 24 and 36 hours in the WT than in the *trif^-/- ^*group; however, it was lower at 12 hours. (**I**) TNF-α concentration was significantly increased in the WT at 24 and 36 hours compared with the *trif^-/- ^*group. (**J**) IL-6 concentration was significantly increased in the WT at 24 and 36 hours compared with the *trif^-/- ^*group. (**K**) IL-17 concentration was significantly increased in the WT at 12, 24, and 36 hours compared with *trif^-/- ^*group. Experiments were performed in triplicate. n = 3, *P <0.05 *vs*. increase relative to the *trif^-/-^*group (WT group at the same time point). β-actin mRNA was used as an internal control.

Statistical analyses were performed to evaluate the differences between experimental and control groups. We used one-way ANOVA (Figures 1A, 2B-D, 3B, C, 4D, E, 5C, E, 6A-K and 7A-K), method and two-way ANOVA (Figure 5B). Data are presented as mean ± SD, with significance was set at *P *< 0.05 (* in figures) and *P *< 0.01 (** in figures). Graphics and calculations were performed using Graph Pad PRISM (version 5.0 GraphPad Inc. La Jolla, CA, USA), and SPSS software (version 15.0; SPSS Inc., Chicago, IL, USA).

## Results

### Expression of TIR-domain-containing adapter-inducing interferon-β in wild-type retinas 1, 3, and 7 days post-crush

TRIF is the unique adaptor of TLR3, which is expressed in microglia and presumably acts as an intracellular TLR-bound molecule. TRIF is important for TLR signal transition [21]. When the ON was injured, TRIF was unregulated from PC day 1-7 in the retina, in a time-dependent manner. At days 3 and 7 PC especially, TRIF expression was significantly higher than in the sham and day 1 PC group independently (Figure 1A, n = 4, *P *< 0.05). Using dual-label immunofluorescence staining, co-expression of TRIF and Iba-1 was detected in microglia but not in neurons or astroglia, indicating that microglia express specific TRIF when the optic nerve is injured (Figure 1B).

### TIR-domain-containing adapter-inducing interferon-β-deficient mice exhibit robust axon regeneration ability

The GAP43 antibody was used to evaluate the newly outgrown axons from soma, as described previously [4], We observed significant axon regeneration in *trif^-/- ^*mice, and *trif^-/- ^*RGCs exhibited robust regenerative ability after lesion, in contrast to WT RGCs (Figure 2A). On day 1 PC, GAP43-labeled axons stopped at the crush site, with no labeled fibers found distal to the crush site, either in the WT or *trif^-/-^*groups. On days 3 and 7, the mean estimated numbers of outgrown axons (100 μm distal to lesion site) were 392 ± 66 and 542 ± 49, respectively (n = 6, Figure 2B, C) in *trif^-/- ^*RGCs, whereas much less axon outgrowth was visible in the WT group (Figure 2A). This result partly correlated with a previous study, which reported observation of numerous axonal sprouts on day 3 PC in phosphatase and tensin homolog (PTEN)-deleted mice [9].

### Axon regeneration and survival in retinal ganglion cells *in vitro *are independent of TIR-domain-containing adapter-inducing interferon-β deficiency

To determine whether the deficiency of TRIF has any effect on the ability of RGCs to promote axon regeneration, RGCs were separated from the retina using serum-free neural basal medium to evaluate the ability of RGC regeneration. Three days after culture, we quantified the mean length of axons positively labeled with GAP43. The mean axon length of RGCs was 14.8 ± 1.3 μm in *trif^-/-^*mice (n = 9) and14.5 ± 1.7 μm in WT mice (n = 9), with no significant difference between the groups (Figure 2D). To evaluate the survival ability, we scratched the cultured RGCs on the plate to mimic the *in vivo *lesion model, and found that there was no difference in the survival ratios of *trif^-/- ^*and WT RGCs (n = 14, *P *< 0.05, data not shown).

### TIR-domain-containing adapter-inducing interferon-β deficiency prevents optic nerve loss

With βIII-tubulin staining, we were able to observe RGCs and optic nerve bundles *in vivo *in whole-mount retinas. The width and density of nerve bundles were significantly different between *trif^-/- ^*and WT mice by day 28 PC. The density of RGCs and the thickness of nerve bundles were higher in the retinas of *trif^-/- ^*mice compared with WT mice (Figure 3A). Using Image Pro Plus software, the width of the nerve bundle was analyzed, with a mean width of 10.38 ± 0.76 μm found in the *trif^-/- ^*group (n = 26) and 4.24 ± 0.81 μm in the WT group (n = 30, Figure 3B). *ρ*_ONB _was 262 ± 18 (n = 26) in the *trif^-/- ^*group, which was greater than that of the WT group (198 ± 3, n = 30, Figure 3C), indicating that ONs without TRIF were resistant to neural atrophy.

### TIR-domain-containing adapter-inducing interferon-β-deficient mice found higher survival rates after optic nerve lesion

Before the lesion operation, RGS axons in the soma were retrograde-labeled with FG in control retina (Figure 4A). The animals bred, and the survival rate was 100%. In the ON lesion groups, fewer RGCs remained visible with FG labeling from day 7-21 PC in both groups (Figure 4B, C). All the labeled RGCs were gold in color, and characteristically round or oval under UV microscopy while they were alive. Quantitatively, the mean number of surviving RGCs on days 7, 14 and 21 PC were 1010 ± 321, 867 ± 151, and 726 ± 89, respectively, in the *trif^-/- ^*retina, (six RGCs per field; *P <*0.05; Figure 4C, D)and 452 ± 98, 326 ± 120, and 312 ± 115, respectively, in the WT group (n = 6; Figure 4B, D). The survival rateswere 69.25 ± 4.03%, 56.75 ± 1.75% and 49.75 ± 3.33%, respectively, for *trif^-/- ^*retina, (Figure 4E), which was higher than those of the WT group (51.75 ± 4.68%, 30.25 ± 3.43%, 25.00 ± 2.80%, n = 6, *P <*0.05), indicating that TRIF deficiency protects the retina from RGC apoptosis or necrosis.

### Optic nerve lesion induced microglial activation in wild-type but not *trif^-/- ^*animals, both *in vivo *and *in vitro*

In the adult retina, ramified microglial cells are found in both the inner and outer plexiform layers. CD11b, a microglial marker, was used to identify the activated and inactivated states of microglia [28]. The results showed that in whole-mount retina immunostaining, the microglia was ramified, surrounded by fine protractions. However, after 7 dPC, the microglia formed a dotted or short ramified shape in the WT retina, but not in the *trif^-/- ^*retina. At 14 d PC, the WT microglia had migrated towards one pole (foramen opticum) with dot or amoeboid shape to the body, whereas the *trif^-/- ^*microglia did not exhibit directivity (Figure 5A). From 14 dPC, the number and density of microglia increased in the sham-operated groups of both *trif^-/- ^*and WT retina (Figure 5B). Statistical analysis indicated that at 7 dPC, the estimated number of activated microglia in was 174 ± 28/mm^2 ^in the *trif^-/- ^*retina and 189 ± 24/mm^2 ^in the WT retina, which had increased to 29 ± 11/mm^2 ^in the *trif^-/- ^*group and 242 ± 32/mm^2 ^by 14 dPC (n = 6, *P *> 0.05, Figure 5B). No significant difference was seen between the *trif^-/- ^*and WT groups at the same time points, but differences were identified between different time points (sham, day 7, and day 14 for both *trif^-/- ^*and WT, *P <*0.05). In addition, there was little difference between the retinas of the *trif^-/- ^*and WT groups at 1 and 3dPC (data not shown).

We examined microglia migration by placing the microglia in the transparent polyester membrane of a transwell plate, with RGCs in the lower well of the plate. On the first day after lesion, we observed axonal outgrowth from the soma in the co-culture group. Meanwhile, in accordance with the axon lesion in the lower well, the upper microglia migrated across the transwell membrane (Figure 5E). The number of migrated migroglia was 217 ± 34/cm^2 ^in the *trif^-/- ^*(n = 6) group and 439 ± 41/cm^2 ^(n = 6) in the WT group, with the *trif^-/- ^*microglia having a lower migration ability than the WT microgliatowards the lesioned RGCs *in vitro*.

**Figure 8:**
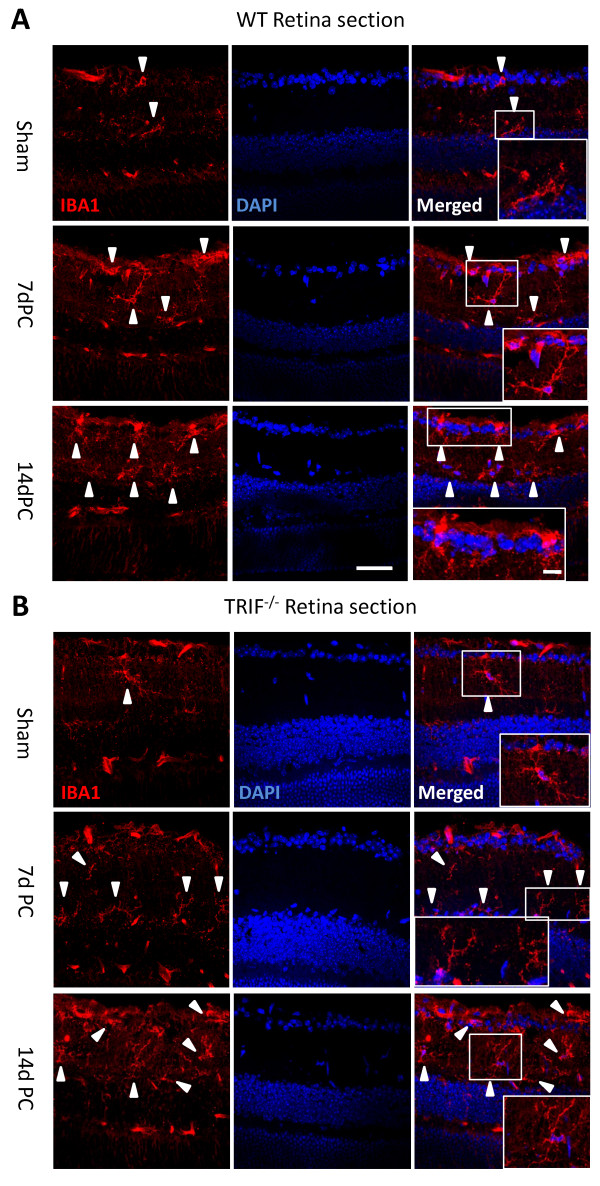
**Microglial cells in retinal sections at post-crush days 7 and 14 (7dPC and 14dPC)**. Microglia in retinal sections were different between the WT and trif-/- groups at 7dPC and 14dPC. **(A)** In the WT group, microglia were located in the ganglion cell layer (GCL) and the inner plexiform layer (IPL). After stimulation by ON injury, more microglia were located in the GCL and IPL, and had a ramified and dotted shape at 7dPC. More microglia with a dotted shape and short processes were located in the GCL and IPL at 14dPC.** (B)** In the trif-/- group, the microglia located in the GCL and IPL had a ramified shape. Scale bar = 20 &#956m. Scale bar (in box) = 10 &#956m. GCL, ganglion cell layer; IPL, inner plexiform layer.

At 7dPC in the WT retinas, Iba-1 was expressed in the inner plexiform layer (IPL) and ganglion cell layer (GCL), but it seemed that fewer microglia migrated into the *trif^-/- ^*retinas in transected sections (Figure 8A, B). The *trif^-/- ^*microglia had more processes, and a ramified shape. At 14 dPC, more microglia had migrated into the GCL and IPL in WT retina than in *trif^-/- ^*retina, and the former had a dotted or short ramified shape, suggesting that TRIF deletion attenuates the microglial activation.

### TIR-domain-containing adapter-inducing interferon-β deficiency attenuates inflammation via TANK-binding kinase (TKK)1/IκB kinase (IKK)ε and nuclear factor (NF)-κB signaling

The activation of microglia suggests that these cells would be responsive to injured RGCs. To assess the relevant downstream signal of TRIF, we determined the expression of TBK1, IKKε, and NF-κB signaling. In a transwell co-culture system, microglial responses to RGC axon lesion mimicked the optic nerve crush model *in vivo*. Time-course studies were performed on *trif^-/- ^*microglia using western blot analysis, and compared with the WT. The protein levels of β-actin (the internal control) remained largely unchanged in both control and stimulated cells. TBK1 was upregulated between 12 hours and 36 hours in the WT group, whereas did the maximum TBK1 expression was reached in the *trif^-/- ^*group by 12 hours. The WT and *trif^-/- ^*groups had different time-dependent behaviors (Figure 7). Accompanying the increase in IKKε expression, NF-κB was increasingly expressed during the period from 0 to 36 hours. However, the *trif^-/- ^*group had significantly different time-dependent behavior; from 12 hours to 36 hours, the expression of NF-κB decreased gradually and IKKε expressed steadily, suggesting that TRIF deficiency limits the activity of downstream molecules, a result consistent with those of Chang *et al. *[29].

### Expression of inducible nitric oxide synthase, tumor necrosis factor-α, interferon-β, and interleukins-1β, 6 and 17 decrease in *trif^-/-^*microglial cells after axonal lesion

To determine whether the decreased expression of TBK1/IKKε and NF-κB proteins leads to changes in inflammatory factors, we next characterized the expression of iNOS, TNF-α, IFN-β, IL-1β, IL-6, and IL-17 by quantitative (q)PCR in microglial cells and the supernatant of conditioned medium that was pre-stimulated by injured RGCs for 12, 24, and 36 hours. The housekeeping gene β-actin and the genes for the inflammatory protein iNOS, TNF-α, IFN-β, IL-1β, IL-6, and IL-17 were amplified for 40 cycles. Expresion of TNF-α, IL-17, and IFN-β mRNAs were significantly lower in the *trif^-/- ^*group than in the WT group, especially for the pre-stimulation group at 36 hours (Figure 6A, C, E). The upregulation of these mRNAs was time-dependent in the pre-stimulated time course. However, in the WT group, there was a marked increase in expression at 36 hours for iNOS, TNF-α, and IFN-β (Figure 6A, C, F), and at 24 hours for IL-6, IL-1β, and IL-17 (Figure 6B, D, E). In the *trif^-/- ^*group, the expression of iNOS and IL-17 did not significantly differ from the control up to 36 hours. However, TNF-α and IL-6 were upregulated at 12 and 24 hours, and then downregulated at 36 hours. The most interesting factor was IL-1β, whose expression reached a maximum (6.8 times that of the WT maximum) at 12 hours and decreased suddenly at 24 hours (43.5% of WT) and 36 hours (46% of WT) in the *trif^-/- ^*group.

To determine the release of inflammatory factors in the microglial cell supernatant that was pre-stimulated with injured RGCs, we performed ELISA detection. Similar to the qPCR results for TNF-α, IFN-β, IL-1β, IL-6, and IL-17 the change in the inflammatory factor levels depended on pre-stimulation time course and TRIF deficiency (Figure 6G-K). In the WT group, release of TNF-α and IFN-β gradually increased from 0 to 36 hours, and were significantly higher than those of the *trif^-/- ^*group at 36 hours. Protein levels of IL-6 and IL-17 were much higher in the WT than the *trif^-/- ^*microglial cells at 24 and 36 hours. By contrast, increased IL-1β was detected at 12 hours in the *trif^-/-^*group but not in the WT group, and it rapidly decreased to a lower level by 24 and 36 hours compared with the WT group.

## Discussion

In the retina, oxidative stress induce by trauma, retinal neovascularization, and sterile inflammation may contribute to various eye diseases, including retinal ischemia and glaucoma [30]. As a CNS neuron, the optic nerve cannot regenerate after injury [1,2], except in certain special situations, such as in the case of oncomodulin stimulation [26], Mst3b-mediating axon regeneration [31], and intrinsic axon regeneration regulated by the Kruppel-like factor family [32]. TLR signaling is crucial for functional recovery after peripheral nerve injury [33] and optic nerve injury [34]. In the present study, we found that TRIF gene ablation exerted a positive effect on the regeneration of the ON, which is a classic model for studying the CNS. Statistical analysis verified that the process of recovery was different between TRIF-sufficient and -deficient groups.

Using GAP43 staining, we found that by 7 dPC, TRIF deficiency exerted a significant effect on longer regenerative axons compared with the WT group, which is similar to the results described by Yin *et al. *[4]. This suggests an unexpectedly powerful neuroprotective effect of TRIF deficiency in microglial cells. One hypothesis to explain this is that in the adult CNS, the capacity for axon outgrowth is reduced by intrinsic factors; however, the molecular nature of this reduction is still unclear [35]. In our results, adult *trif^-/- ^*mice had the ability to regenerate axons in the ON. However, the *in vitro *results showed that *trif^-/- ^*RGCs cultured solely with serum-free medium had the same limited regeneration ability as WT RGCs (Figure 2D). In addition, TRIF was not expressed in WT RGCs. The results indicated that TRIF is not an inhibitory molecule that limits the regenerative ability of retinal axons. GAP43 is a membrane phosphoprotein that is normally undetectable in the mature ON, but is strongly expressed in axons undergoing regeneration [36]. In adult mice, we found that *trif^-/- ^*RGCs were unable to regenerate axons on their own, without interaction with the microenvironment.

The *in vivo *ON lesion model and the *in vitro *RGC culture produced different results for the regenerative ability of WT and *trif^-/- ^*RGCs, indicating that the microenvironment plays some role in the regenerative ability of the RGCs. To explore the effect of TRIF, we used a dual-label immunochemistry method on retinas. We found that astrocytes and neurons did not express TRIF, but microglia did (Figure 1B). As a downstream adaptor of TLR4, TRIF deletion may contribute to the survival of RGCs by microglial inactivation to some extent. A similar neurotoxic role for microglia-mediated fundamental injury or repair was described by Nguyen *et al. *[37]. Recently, TLR4, MyD88, or TICAM1 ablation were reported to promote proliferation in the post-natal mammalian retina [37]. However, no study has reported that TRIF deletion promotes axon regeneration of adult RGCs by microglial inactivation. Therefore, our results provide some new data for neuroimmunological and neuroinflammatory aspects.

Recent studies have identified novel roles for TLRs in the CNS and peripheral nervous system [38]. Downstream of TLR3 and TLR4, activation of TRIF is essential for the MyD88-independent pathway [39]. Similarly, both IFN-β mRNA and protein were reduced in the *trif^-/- ^*compared with the WT microglia when stimulated by RGC lesions *in vitro*. IFN-β is one of the factors released by microglia, and is used as a clinical treatment for prevention of relapse in all subtypes of multiple sclerosis (MS) [40]. However, it may severely exacerbate optic-spinal MS in the neuromyelitis optica spectrum, amplifying CNS inflammation, and exacerbating the disease [40-43]. To our knowledge, IFN-β was reduced in the study, allowing promotion of RGC axon regeneration by TRIF deficiency and a neutralizing antibody, which supports the work of Shimizu *et al. *[42]. IFN-β is a factor released downstream, and is activated by an intracellular mechanism and upstream receptors in microglia. Several lines of evidence suggest that upstream of the pro-inflammatory release, the microglial innate immunological responses are involved in microglial activation [44,45]. Furthermore, there remains a possibility that TRIF deficiency may contribute to IFN-α delivery, and the release of IFN-β may trigger other pro-inflammatory genes that have dual roles in benefiting or impairing neurons during different time periods, exerting a beneficial or detrimental effect on the retina and ON regeneration. Thus, a further challenge is to clarify other upstream and intracellular mechanisms, for example TLR3 or TLR4 signaling, and IF3 activation.

As described previously [46,47], overexpression of TRIF causes activation of the NF-κB promoter in 293 cells and the IFN-β promoter. In our study, expression of TRIF gradually increased at 1, 3 and 7 dPC in the WT group (Figure 1A). Compared with the WT group, expression of TBK1, IKKε, and NF-κB decreased in the *trif^-/- ^*group, indicating that the microglial activation of TBK1, IKKε, and NF-κB in response to pre-stimulation by injured RGCs is TRIF-dependent. TRIF-mutant mice were defective in TLR3- and TLR4-mediated inflammatory responses [48], supporting our observation that TRIF deficiency limits the inflammatory effect on RGCs via TBK1, IKKε, and NF-κB signaling pathways. However, other signaling pathways may be involved in the activation of NF-κB signaling, which needs further investigation.

IL-1β output depends on the activation of NF-κB, leading to some neurotoxicity in the CNS [49]. In our results, the *trif^-/- ^*group had higher IL-1β expression in the early phase of stimulation compared with the WT group, but at a later stage, this decreased suddenly. This is an early-phase activation by MyD88 compensation. The pulse, not the constant release of IL-1β, which decreased significantly at 24 and 36 hours, may not significantly influence the survival of RGCs.

IL-6 and IL-17 expression was different between WT and *trif^-/- ^*microglial cells pre-stimulated by injured RGCs. IL-6 belongs to the neuropoietic cytokine family, and is a multifunctional cytokine that regulates cell differentiation, growth, and survival in a variety of diseases [50,51]. Induced IL-6 accompanied by TNF-α and IFN-γ probably contributes to the lower toxicity seen with conditioned medium collected from retinas incubated with the Rho-kinase inhibitor H1152p [52]. Microglial IL-17 is produced in response to IL-23 and IL-1β, and contributes to autoimmune diseases in the CNS [53]. Similarly, we found that in a co-culture system of microglial cells and RGCs, the mRNA and protein levels of TNF-α, IL-6, and IL-17 were significantly higher in the WT group than in the *trif^-/- ^*group. This suggests that microglial TRIF gene deletion induces fewer neurotoxic factors and inflammatory responses with harmful effects on axonal regeneration. A previous study by Sivakumar *et al. *[54] identified microglial inflammation in a hypoxic neonatal retina model, which is consistent with our results, performed in a co-culture system of microglial cells and RGCs.

The appearance of the microglia in the healthy mature tissues of brain, spinal cord, and retina suggest both a similarities and dissimilarities between these tissues. CD11c^+^CD45^lo^F4/80^+ ^cells in retina have been identified as microglia, which were considered initially to have antigen-presenting capacity both in retina and in brain [55]. Similar to brain, retinal microglia express IL-27 and IL-10 to control the severity or duration of inflammation in the CNS [56]. In response to injury, the immunoproteasome is significantly upregulated in both retina and brain. However, the expression of the immunoproteasome, which generates immunogenic peptides for antigen presentation, is approximately twofold higher in retina than in normal brain [57,58]. In scrapie-induced neurodegeneration, the activation and function of microglia under the control of the PrP promoter (tg7) and the neuron-specific enolase promoter (tgNSE) are clearly different in the retina and brain [59].

With regard to human disease, traumatic optic neuropathy (TON) is one of the most common neuropathies, affecting an increasing number of people worldwide and leading to loss of neural cells of the eyes. Although recent advances in treatment can now slow its progression, many people with TON still experience an irreversible loss of vision [60,61]. ON and retinal research may provide insights into CNS disease. Regulation of inflammation could provide strong evidence for attenuating the injury to protect the ON and retina from neuropathy.

Upstream TRIF signaling is involved in the initiation of inflammatory factor release, which activates and recruits microglia in response to RGC axon injury via the TBK1-IKKε-NF-κB signaling pathways. Overexpression of TRIF and NF-κB is likely to induce neurotoxicity [46,47].

## Conclusions

In summary, our findings suggest a specific upstream target for potential therapeutic interventions aiming at inhibition of TRIF-induced inflammatory responses. TRIF deficiency results in protection of neurons from microglial neurotoxicity, attenuates the release of inflammatory factors, and promotes axon regeneration. As innate immunity is involved in various neurodegenerative diseases, further investigation of novel treatment strategies that interfere with the activation of inflammatory responses after retinal injury remains an important area of research.

## Abbreviations

BSA: bovine serum albumin; CNS: central nervous system; DAPI: 4', 6-diamidino-2-phenylindole; DMEM: Dulbecco's Modified Eagle's Medium; ELISA: enzyme-linked immunosorbent assay; GADPH: GAP: growth-associated protein; GCL: ganglion cell layer; HMG: high mobility group protein; IFN: interferon; IKK: IκB kinase; IL: interleukin; iNOS: inducible NO synthase; IP: intraperitoneal; IPL inner plexiform layer; IRF: interferon regulatory factor; KO: knockout; MyD: myeloid differentiation factor; nitric oxide; NF-κB: nuclear factor-κB; NO: nitric oxide; ON: optic nerve; PBS: phosphate-buffered saline; PCR: polymerase chain reaction; PFA: paraformaldehyde; PTEN: phosphatase and tensin homolog; RGC: retinal ganglion cell; RIP: receptor-interacting protein; TANK: tumor necrosis factor receptor-associated factor family member-associated nuclear factor-κB activator; TBK: TANK-binding kinase; TKK: tumor necrosis factor receptor-associated factor family member-associated nuclear factor-κB activator-binding kinase 1; TNF: tumor necrosis factor; TRIF: Toll-interleukin-1 receptor-domain-containing adapter inducing interferon beta; WT: wild-type.

## Competing interests

The authors declare that they have no competing interests.

## Authors' contributions

This study was based on the original idea of SL, SL, and BS. SL, CB, and HZ carried out the immunostaining and molecular biology studies. SL and YL drafted the manuscript, and JZ and YX proofread the manuscript. SL and QG carried out the data analysis. SL and BS were responsible for supervising the experiments, data analyses, and writing of the manuscript. All authors read and approved the final manuscript.
