# Correction: Microglial TIR-domain-containing adapter-inducing interferon-β (TRIF) deficiency promotes retinal ganglion cell survival and axon regeneration via nuclear factor-κB

**DOI:** 10.1186/1742-2094-10-48

**Published:** 2013-04-19

**Authors:** Sen Lin, Yajie Liang, Jiqiang Zhang, Chen Bian, Hongli Zhou, Qiang Guo, Ying Xiong, Shurong Li, Bingyin Su

**Affiliations:** 1 Department of Neurobiology, Chongqing Key Laboratory of Neurobiology, Third Military Medical University, Chongqing, 400038, PR China; 2 Department of Histology and Embryology and Neurobiology, Development and Regeneration Key Laboratory of Sichuan Province, Chengdu Medical College, Chengdu, 610083, PR China; 3 Department of Pathology, Chengdu Medical College, Chengdu, 610083, PR China

## Correction

After publication of the original article [1] it was brought to the attention of the publishers that several errors were introduced during the typesetting and production process. In the original article Figures six (Figure 1 here), seven (Figure 2 here) and Figure eight (Figure 3) do not correspond to their legends and some typographical errors were introduced into the legends. The publishers regret any inconvenience this may have caused.

**Figure 1:**
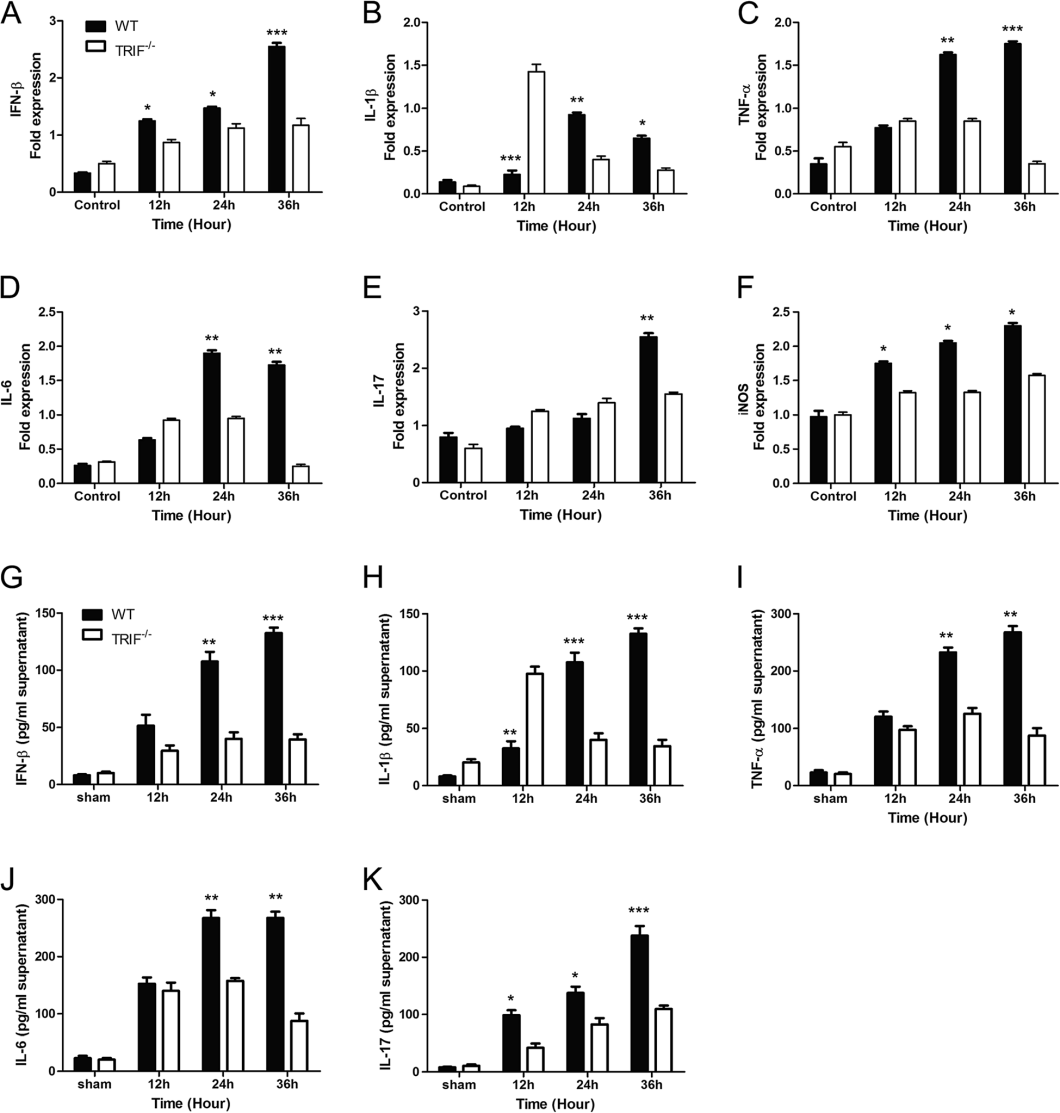
**[Updated Figure 6 from original manuscript]. TIR-domain-containing adapter-inducing interferon-β (TRIF) deficiency attenuates microglial inflammatory factor release.** Real-time reverse transcriptase (RT)-PCR and ELISA results of wild-type (WT) and *trif*^*-/-*^ microglia pre-stimulated by injured RGCs in a transwell system to identify changes of inflammatory factors. (**A-F**) Measured by real-time RT PCR; **(G-K)** measured by ELISA. (**A**) Bar graphs showing interferon (IFN)-β mRNA expressed in the control, and at 12, 24, and 36 hours in WT and *trif*^*-/-*^ microglia by real-time RT-PCR. Significant differences were seen at 36 hours in microglia pre-stimulated with injured RGCs. (**B**) Interleukin (IL)-1β mRNA expressed in the WT and *trif*^*-/-*^ groups. At 12 hours, IL-1β mRNA expression was higher in the *trif*^*-/-*^ than in the WT group; however, at 24 and 36 hours, it was higher in the WT than in the *trif*^*-/-*^ group. (**C**) At 24 hours and 36 hours, tumor necrosis factor (TNF)-α was upregulated to a greater extent in the WT group. (**D**) IL-6 was significantly higher in the WT than in the *trif*^*-/-*^ group at 24 and 36 hours. (**E**) IL-17 was upregulated at 36 hours in the WT. (**F**) Inducible nitric oxide synthase (iNOS) increased from 12 to 36 hours in the WT. Similar to the PCR results, (**G**) IFN-β release increased markedly at 24 and 36 hours. (H) IL-1β had a greater increase at 24 and 36 hours in the WT than in the *trif*^*-/-*^ group; however, it was lower at 12 hours. (**I**) TNF-α concentration was significantly increased in the WT at 24 and 36 hours compared with the *trif*^*-/-*^ group. (**J**) IL-6 concentration was significantly increased in the WT at 24 and 36 hours compared with the *trif*^*-/-*^ group. (**K**) IL-17 concentration was significantly increased in the WT at 12, 24, and 36 hours compared with *trif*^*-/-*^ group. Experiments were performed in triplicate. n = 3, *P <0.05 *vs*. increase relative to the *trif*^*-/-*^group (WT group at the same time point). β-actin mRNA was used as an internal control.

**Figure 2:**
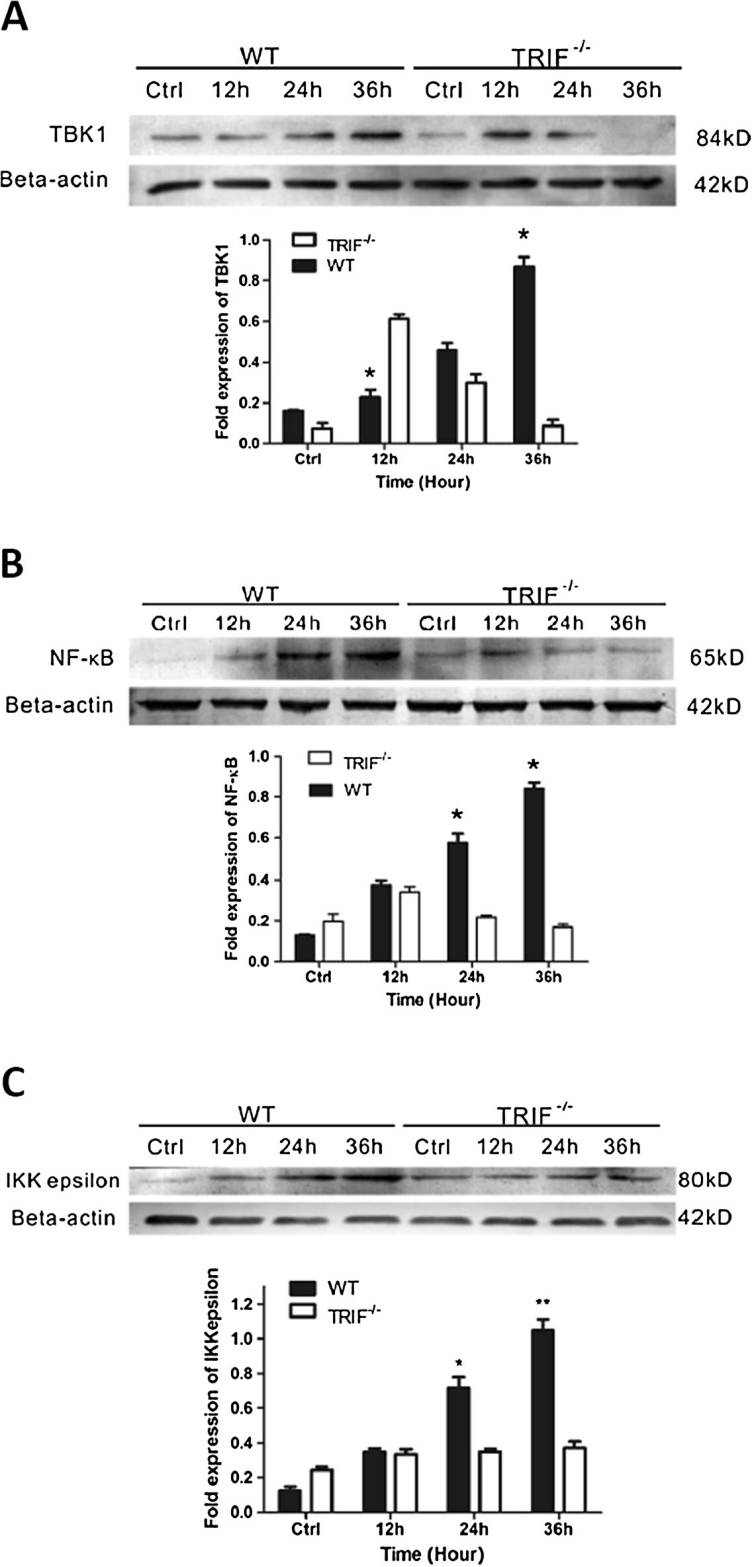
**[Updated Figure 7 from original manuscript]. TIR-domain-containing adapter-inducing interferon-β (TRIF) deficiency attenuates inflammation via TANK-binding kinase (TBK)1/ IκB kinase (IKK)ε and nuclear factor (NF)- κB signaling.** Western blot results for wild-type (WT) and *trif*^*−/−*^ microglia pre-stimulated by injured retinal ganglion cells (RGCs) in a transwell system identifies the signaling changes downstream of TRIF. (**A**) Bar graph showing that TBK1 was upregulated gradually in the WT group; however, *trif*^*−/−*^ effectively suppressed TBK1 from 24 to 36 hours. (**B**) *Trif*^*−/−*^ effectively suppressed NF-κB from 12 to 36 hours. (**C**) Bar graph showing that *trif*^*−/−*^ effectively suppressed IKKε from 12 to 36 hours. *P<0.05, **P<0.01 *vs.* WT group at the same time point. β-Actin was used as an internal control.

**Figure 3:**
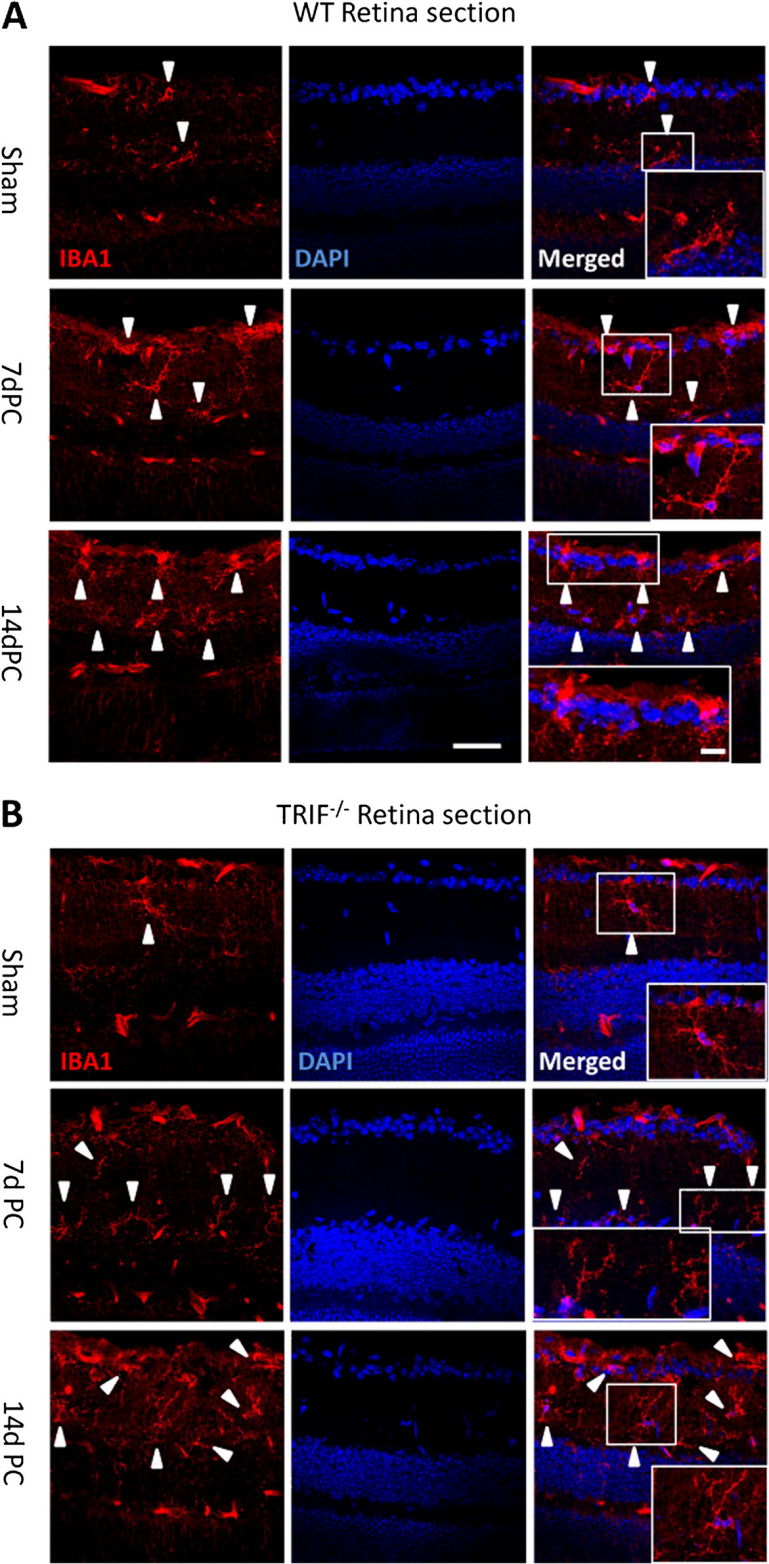
**[Updated Figure 8 from original manuscript]. Microglial cells in retinal sections at post-crush days 7 and 14 (7dPC and 14dPC).** Microglia in retinal sections were different between the WT and *trif*^−/−^ groups at 7dPC and 14dPC. (**A**) In the WT group, microglia were located in the ganglion cell layer (GCL) and the inner plexiform layer (IPL). After stimulation by ON injury, more microglia were located in the GCL and IPL, and had a ramified and dotted shape at 7dPC. More microglia with a dotted shape and short processes were located in the GCL and IPL at 14dPC. (**B**) In the *trif*^*−/−*^ group, the microglia located in the GCL and IPL had a ramified shape. Scale bar = 20 μm. Scale bar (in box) = 10 μm. GCL, ganglion cell layer; IPL, inner plexiform layer.

It was also noticed by the authors that the image they provided for Figure four panel C (Figure 4 here) was incorrect. The authors apologize for any inconvenience this may have caused.

**Figure 4:**
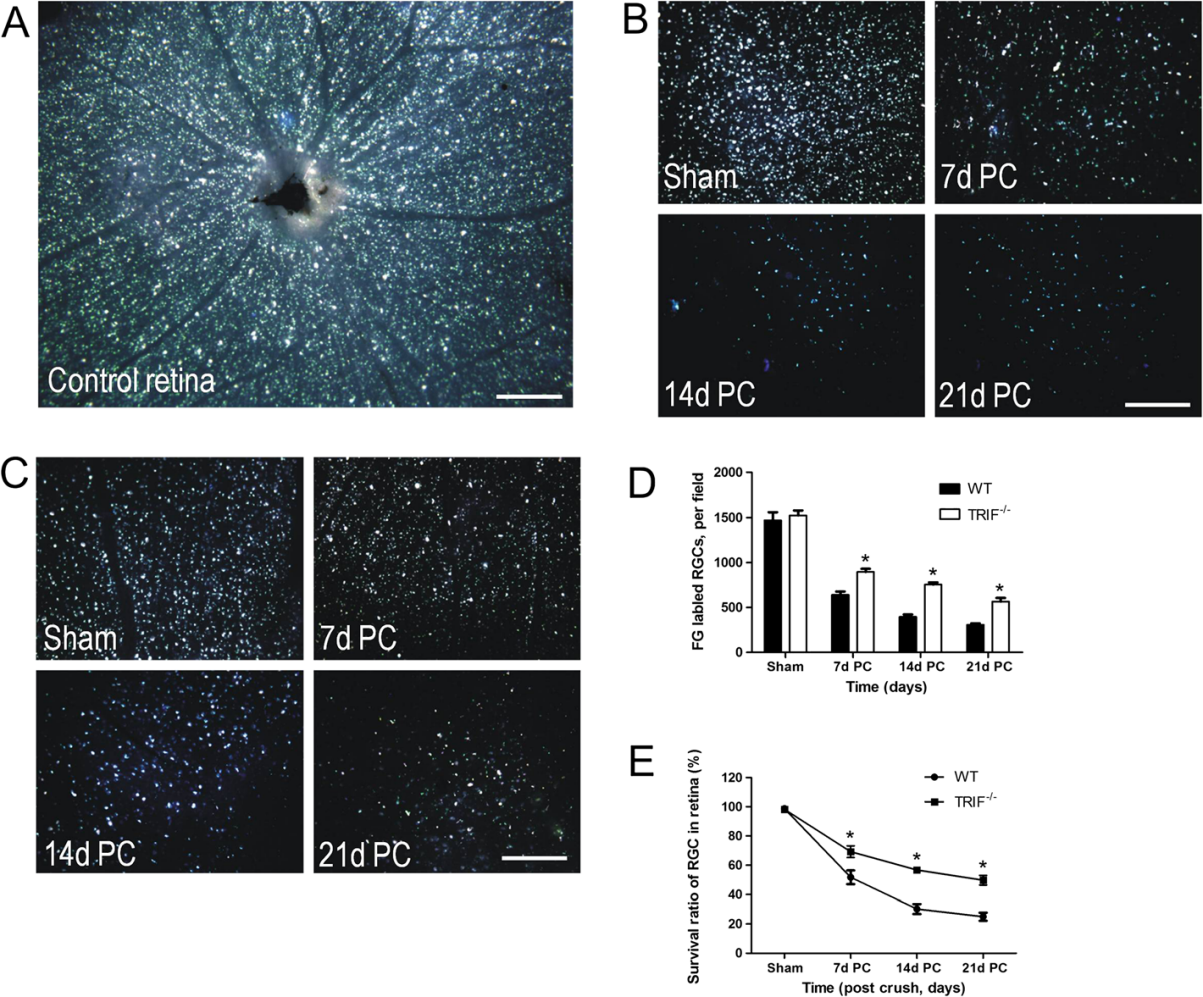
**[Updated Figure 4 from original manuscript]. Retinal retinal ganglion cell (RGC) survival detected by Fluorogold (FG) retrograde labeling.** (**A**) FG was transported retrograde to RGC soma in a whole-mount retina used as a control. From 7 to 21 days post-crush (7dPC to 21dPC, FG-labeled RGCs declined in (**B**) the WT and (**C**) *trif*^*-/-*^ groups. Scale bar, 100 μm. The number of labeled RGCs was analyzed to confirm that more RGCs survived in the *trif*^*-/-*^ group than in the WT group (**D**). *P < 0.05 vs. WT group. (**E**) Survival ratio of RGCs in *trif*^*-/-*^ group was higher than that in the WT group from 7dPC to 21dPC. *P < 0.05 vs. WT group.
